# Emulsions, and How to Make Them

**Published:** 1882-05

**Authors:** C. Lewis Diehl


### SELECTIONS.

### EMULSIONS, AND HOW TO MAKE THEM.

Among the many preparations that manufacturers of pharmaceutic specialties have made much capital out of in recent years, emulsions of cod-liver oil take a very prominent place. From the claims set forth in their advertisements—which may be either by label. by circular, or by the personal interview of the manufacturer's agent—one is easily led to believe that not alone superior knowledge and skill are requisite in their production, but also that their successful production is possible only with the aid of expensive machinery and the advantages that are derived from abundant capital and a particularly favored market. Yet nothing is farther from the truth ; for, while it is not disputed that a certain amount of knowledge and skill are required, that machinery is a necessity to the production of emulsions in large quantities, and that capital and a good market are advantageous in a commercial sense, their production requires no more knowledge and not as much skill as does the compounding of most of the prescriptions daily put up in our pharmacies, while all the apparatus necessary are a mortar and pestle, and accurate implements of measurement. These, with good, fresh cod liver oil—which can be obtained without difficulty—make the production of an emulsion as easy an operation as the compounding of the simplest prescription. Moreover, such emulsions possess real advantages over the manufactured article. They are, in the first place, perfect emulsions, which the commercial products in many cases are not. Then, being prepared at the time when they are prescribed, they are absolutely fresh ; and there is perhaps no class of preparations in which this is more desirable. It is true the manufacturers usually claim that their emulsions keep well ; but at the same time they are very careful to recommend that such be kept in a cool place. The truth is that, notwithstanding such care the preparations furnished soon undergo change, become unsightly and unfit for use ; and if it is further considered that this care in their preservation cannot always be observed, and, as a rule, is disregarded both by the wholesaler and retailer, it cannot be wondered that manufactured emulsions are often dispensed in a very unsatisfactory condition.

These considerations have suggested to me the propriety of offering some formulas for emulsions, in the hope that, in the absence of authoritative formulas, physicians may find them of sufficient merit to adopt them in their practice ; giving them at the same time the assurance that careful pharmacists following them will succeed as well as I have succeeded in preparing them.

The successful formation of emulsions, whether of fixed or volatile oils, is dependent upon certain rules well understood by accomplished pharmacists, which, when deviated from, will invariably embarrass the operator, either by retarding or completely preventing perfect emulsification. These rules are :

1. That the water and gum arabic* shall be in definite and absolute proportion to each other. This proportion is three (3) parts of water to two (2) parts of gum, both by weight.

2. That the relation of oil to gum (and water) shall be definite within certain limits—that is to say, the mucilage formed in the above proportions is capable of perfectly emulsifying a minimum and a maximum proportion of oil. The minimum proportion is two (2) parts of oil to one (1) part of gum ; the maximum proportion is four (4) parts of oil to one (1) part of gum.

3. That the trituration of the oil, gum, and water be continued till a perfectly homogeneous, milky white, thick, creamy, mixture is formed —*i.e.*, until perfect emulsification takes place—before the addition of a further quantity of water or other liquid.

The thick, creamy emulsion obtained, if the above conditions are fulfilled, must be the basis of all perfect emulsions. It will bear dilution to any extent with water, forming mixtures, varying, according to the proportion added, from the appearance and consistence of cream to that of very thin milk. Obviously the water may be substituted by solutions of saline compounds, syrups, etc., and this enables the production of the various combinations of cod-liver oil in current use from the above thick, creamy emulsion, which for distinction I shall designate as—

I. *Concentrated Emulsion of Cod-Liver Oil.*—Take of fresh Norwegian cod-liver oil eight (8) troy ounces ; powdered gum arabic, two (2) troy ounces ; distilled water, three (3) troy ounces. First weigh the gum into a wedgewood or porcelain mortar, then the oil, and triturate till the

* The writer is well aware that other emulsifying agents have been proposed and are used, but he is satisfied that none of these answer as well as does gum arabic.

gum is well mixed with the oil ; then weigh into the mixture the distilled water, and triturate the whole briskly until the mixture thickens and acquires a pasty consistence and milky whiteness. Now scrape down the portions adhering to the sides of the mortar and to the pestle, and continue the trituration for a short time, after which add such other ingredients as may be desirable, or transfer the concentrated emulsion to a wide-mouthed bottle for future use.

This concentrated emulsion will keep for a reasonable time in cold weather, and, if placed in the ice-chest, also during warm weather. It may therefore be kept in stock if the demand for emulsions is brisk enough to justify it ; but, inasmuch as its preparation does not consume more than five or ten minutes, it is advised to always prepare it fresh, or, at all events, never to prepare more than a week's supply, particularly in summer. Its consistence is such that it is poured out of the containing vessel with difficulty ; hence the necessity of using one with a wide mouth, which should be as securely stoppered as possible, and should be cleaned very carefully each time it is refilled. All this takes time and involves trouble, which is prevented by preparing the concentrated emulsion only as required.

II. *Simple Emulsion of Cod-Liver Oil.*—Take of concentrated emulsion of cod-liver oil thirteen (13) troy ounces ; oil of wintergreen, twenty-four (24) drops ; syrup, one (1) fluid ounce ; water, three (3) fluid ounces. Weigh the concentrated emulsion into a mortar, add the oil of wintergreen, and triturate thoroughly ; then gradually add first the water, and then the syrup.

The manipulation for this emulsion is typical for all the other cod-liver oil emulsions given below. It has the consistence of very thick cream, but is readily poured out of narrow-mouthed bottles, is milky white, and mixes readily with water or other liquids that may be administered with it. It contains exactly fifty per cent (by volume) of oil, the quantity that manufactured emulsions are said to contain, although I have convinced myself that some of them do not contain that proportion. The oil of wintergreen disguises the odor of the cod-liver oil very admirably, and has the further advantage that it acts as a preservative.

III. *Emulsion of Cod-Liver Oil with Hypophosphite of Lime.*—This differs from the simple emulsion in that one hundred and twenty-eight grains of hypophosphite of calcium are dissolved in the water, each tablespoonful of the finished emulsion containing four grains of that salt.

IV. *Emulsion of Cod-Liver Oil with Hypophosphite of Lime and Soda.*—

This differs from the simple emulsion in that one hundred and twenty-eight grains of hypophosphite of calcium and ninety-six grains of hypophosphite of sodium are dissolved in the water, each tablespoonful of the finished emulsion containing four grains of the calcium and three grains of the sodium salt.

V. *Emulsion of Cod-Liver Oil with Hypophosphites.*—This differs from the simple emulsion in that one hundred and twenty-eight grains of hypophosphite of calcium, ninety-six grains hypophosphite of sodium, and sixty-four grains of hypophosphite of potassium are dissolved in water; each tablespoonful containing four grains of the calcium, three grains of the sodium, and two grains of the potassium salt, and corresponding to a teaspoonful of Churchill's syrup of the hypophosphites.

VI. *Emulsion of Cod-Liver Oil with Phosphate of Lime.*—This differs from the simple emulsion in that two hundred and fifty-six grains of phosphate of calcium are dissolved in water by the aid of one hundred and twenty-eight grains of hydrochloric acid;* each tablespoonful containing eight grains of the phosphate held in pleasantly acid solution.

VII. *Emulsion of Cod-Liver Oil with Phosphate of Lime and Soda.*—This differs from the simple emulsion in that two hundred and fifty-six grains of phosphate of calcium and sixty-four grains of phosphate of sodium are dissolved in the water acidulated with one hundred and twenty-eight grains of hydrochloric acid; each tablespoonful containing eight grains of the calcium and two grains of the sodium salt.

VIII. *Emulsion of Cod-Liver Oil with Lactophosphate of Lime.*—This differs from the simple emulsion in that two hundred and fifty-six grains of lactate of calcium dissolved in two fluid ounces of diluted phosphoric acid are substituted for two fluid ounces of the water, each tablespoonful containing eight grains of lactate of lime, or about ten grains of lactophosphate.

IX. *Emulsion of Cod-Liver Oil with Wild-cherry Bark.*—This differs from the simple emulsion in that the oil of wintergreen is substituted by eight drops of oil of bitter almonds, and in that one fluid ounce of the fluid extract of wild-cherry bark is substituted for one fluid ounce of the water; each tablespoonful containng fifteen minims of the fluid extract and one-fourth of a drop of oil of bitter almonds.

Other combinations of cod-liver oil with different medicinal agents

* The use of hydrochloric acid instead of phosphoric acid is preferred, because the large quantity of the latter required would make the preparation unpleasantly sour.

may be effected in the same way as pointed out in the above, or the proportions of salts may be varied to suit particular cases. The process for the concentrated emulsion also may be applied to the emulsification of other oils, as, for instance, in the following :

X. *Emulsion of Castor-Oil.*—Take of castor-oil four (4) troy ounces ; powdered gum arabic, one (1) troy ounce ; distilled water, one and one half ($1\frac{1}{2}$) troy ounce ; syrup, cinnamon-water, of each three (3) fluid ounces ; spirit of cinnamon, twelve (12) minims. Emulsify the oil with the gum and distilled water as directed under I., then add the other ingredients successively with constant trituration. This emulsion contains thirty-three per cent of castor-oil, and is consequently more limpid than the fifty per cent cod-liver oil emulsions above described, and is in every respect an elegant preparation.—*C. Lewis Diehl, in Louisville Med. News.*

